# Crime rates and sedentary behavior among 4^th ^grade Texas school children

**DOI:** 10.1186/1479-5868-5-28

**Published:** 2008-05-14

**Authors:** H Shelton Brown, Adriana Pérez, Gita G Mirchandani, Deanna M Hoelscher, Steven H Kelder

**Affiliations:** 1 Michael & Susan Dell Center for Advancement of Healthy Living, Division of Management, Policy and Community Health, University of Texas School of Public Health, 313 E. 12th Street, Suite 220, Austin, TX 78701, USA; 2 Department of Bioinformatics and Biostatistics, School of Public Health and Information Sciences, University of Louisville, 555 S. Floyd Street, Suite 4026, Louisville, KY 40292, USA; 3 Family Health Research and Program Development, Texas Department of State Health Services, Austin, TX 78756, USA; 4 Michael & Susan Dell Center for Advancement of Healthy Living, Division of Behavioral Science, University of Texas School of Public Health, 313 E. 12th Street, Suite 220, Austin, TX 78701, USA; 5 Michael & Susan Dell Center for Advancement of Healthy Living, Division of Epidemiology, University of Texas School of Public Health, 313 E. 12th Street, Suite 220, Austin, TX 78701, USA

## Abstract

**Introduction:**

Although per capita crime has generally fallen over the period which coincides with the obesity epidemic, it has not fallen uniformly across communities. It also has not fallen enough to allay fears on the part of parents. Over the past 30 years, technological changes have made the indoor alternatives to playing outside, where children are more vulnerable to criminal activity, more enjoyable (cable TV, video games, and the internet) and comfortable (the spread of air conditioning to low income neighborhoods). We determined whether indoor sedentary behavior patterns are associated with community crime statistics. 4^*th *^graders in the U.S. are typically 9 or 10 years old.

**Methods:**

We used data from the 2004–2005 Texas School Physical Activity and Nutrition (SPAN) survey linked with U.S. Department of Justice, Office of Justice Programs, Bureau of Justice Statistics data for the years 2000 through 2005 and Texas State data on sexual offenders. The probability-based sample included a total of 7,907 children in grade four. Multistage probability sampling weights were used. The dependent variables included were hours of TV watching, video game playing, computer use and total indoor sedentary behavior after school. Incremental Relative Rates were computed for community crime rates including robberies, all violent crimes, murders, assaults, property crimes, rapes, burglaries, larcenies and motor vehicle thefts as well as for sexual offenders living in the neighborhood. The neighborhood refers to the areas where the students at each school live. In the case of sexual offenders, sexual offenders per capita are estimated using the per capita rate in the zip code of the school attended; all other crime statistics are estimated by the crimes per capita in the police department jurisdiction covering the school attended. After controlling for sex, age, and African-American and Hispanic, cross-sectional associations were determined using multivariate Poisson regression.

**Results:**

4^*th *^grade boys were more likely to play video games in communities with increased per 100 population rates of larceny and burglary as well as in communities with increased per capita sexual offenders; 4^*th *^grade girls were more likely to watch television in communities with increased per capita sexual offenders. While 4^*th *^grade girls were more likely to watch TV in communities with increased per capita sex offenders, they were less likely to use computers. Per capita sexual offenders were negatively related to computer use amongst 4^*th *^grade girls.

**Conclusion:**

By combining community crime and cross-sectional individual level data on indoor sedentary behavior, we found that there is an association between community crimes/sex offender rates and certain types of indoor sedentary behavior. The development of technologies in recent decades which makes supervising children easier indoors, where children are much less vulnerable to crime, may be contributing to the epidemic of childhood obesity.

## Background

The prevalence of obesity [1] among children has doubled in the last twenty years [2], disproportionately affecting minorities [3-6]. Part of the problem is the lack of physical activity among youth, which is associated with higher rates of obesity [7-13] Time spent at indoor play comes at the expense of time spent at outdoor play, which may be more physical. Resultingly, sedentary behavior has been shown to be associated with higher rates of child and adolescent obesity [14-18]. During the childhood obesity epidemic, new technologies have made supervising children indoors, where children are less vulnerable to crime in comparison to playing outdoors, both easier and more comfortable. Twenty-five years ago, there were few households with cable television, video games, and no households with internet connections. Many households, especially in low income areas, did not have air conditioning. Technologies which improve the alternatives to playing outside have greatly improved and spread into low income neighborhoods.

Over the period corresponding to the childhood obesity epidemic, crime rates have fallen in the U.S [19]. However, crime has not fallen in all communities and neighborhoods uniformly and many still have very high rates. At the same time, the ease of supervising children indoors, where children are less vulnerable to crime, has improved markedly. Thirty years ago, the options for television viewing after school were limited to local broadcast channels. From 1970 to 2007, the percentage of households with cable grew from 6.7 percent to 64.1 percent [20]. Video game development was in its infancy during the 1970s and personal computing was limited to only the most technologically savvy kids whose parents were wealthy enough to afford the technology. The internet was used by only 15 percent of the population in 1995, but that grew to 73 percent in 2006 [21]. Further, many homes were without air conditioning 30 years ago [22]. Playing indoors has become more and more attractive every year for children and parents/guardians alike.

Physical activity, which is the alternative to sedentary behavior, has been shown to be negatively associated with neighborhood crime and road safety [23]. Weir, Etelson, and Brand have shown that parental perceptions of crime in inner cities is negatively associated with lower physical activity among youth [24]. Road safety and perceptions about the trustworthiness of neighbors have been shown to be positively associated with physical activity among youth [25,26] Access to safe play environments, such as at schools, has been shown to be positively associated with increased physical activity [27]. It's important to note, all of these studies focus on parental perceptions, rather than objective statistics about crime rates. In the one exception, violent crime densities were negatively associated with girl's physical activity [28]. In this paper, we look at a range of crime statistics, including the prevalence of convicted sexual offenders in the area, and relate them to after school indoor electronic entertainment.

Other recent studies have emphasized the effect of contextual variables on health outcomes [29,30]. However, none have examined crime and indoor sedentary activity. Our research, while related to the contextual literature, differs in that we examine a specific health behavior which could be directly attributable to a neighborhood characteristic rather than related to a general health outcome measure. Although childhood obesity measures could ultimately be related to neighborhood crime through their effect on physical activity, obesity is determined by a combination of behaviors including diet. Therefore, instead of focusing on obesity as an outcome, we focus on an important obesity-related behavior which is potentially directly related to neighborhood crime: indoor sedentary behavior.

The purpose of this study is to examine cross-sectional associations between indoor activities, namely computer use, video game playing, and television viewing, with community crime statistics for 4^*th *^grade students in Texas. 4^*th *^graders in the U.S. are typically 9 or 10 years old. Inexpensive technologies which promote sedentary indoor activities are abundant. We therefore hypothesize that community crime rates are positively associated with sedentary indoor activities among 4^*th*^-graders in Texas. The community crime statistics are robberies, all violent crimes, murders, assaults, property crimes, rapes, burglaries, larcenies and motor vehicle thefts. The number of released sexual offenders living in the area is also hypothesized to be positively associated with sedentary indoor activities.

## Methods

The data used were the School Physical Activity and Nutrition III (SPAN). SPAN is a child obesity surveillance system developed and implemented by the University of Texas School of Public Health with support from the Texas Department of State Health Services during the 2004–2005 school year. As part of the SPAN project, student's eating behaviors, nutrition knowledge, and physical activity behaviors were surveyed. SPAN employed a stratified, multistage probability sample of public school children in Texas that included representative samples of White/other, Black or African-American, and Mexican-American-Latino or Hispanic youth. A full description of the SPAN study design and its participants has been documented for the first wave [31], and a brief summary is provided here. In 2004–2005 academic year, Texas was divided into 11 administrative Health Service Regions (HSR) (For a full description, see [32]). Schools which had fewer than 75 students or were charter, magnet or special schools were excluded from the sampling frame. Each HSR was divided into three strata: urban center, other urban/suburban and rural [33]. Schools were randomly selected from HSR or from school districts depending on the strata and then at least two classes. We focused on the 4^*th *^grade because children in the 8^*th *^and 11^*th *^grades are much more independent. The sample size was 7,907, representing the 4^*th *^grade population of 248,838. Approval for the SPAN study was obtained from the Committee for the Protection of Human Subjects at the University of Texas Health Science Center at Houston, as well as the Texas Department of State Health Services Institutional Review Board and participating school districts. Parents completed either an active or passive consent (depending on school district procedures for parental consent), and children completed a child assent form.

SPAN surveys were administered using standard protocols for recalled physical activity. Sedentary behavior was assessed by asking about the amount of the specific behavior on the previous day. The question was, 'Yesterday, how many hours did you watch TV or video movies away from school?'. Sedentary behavior was also assessed by asking about the amount of the specific behavior on an average day. One question was 'How many hours per day do you usually spend on the computer away from school?' (Time on the computer included time spent surfing the internet and instant messaging [on mobile or cell phones].). The other was 'How many hours per day do you usually spend playing video games like Nintendo, Sega, Play Station, Xbox, GameBoy or arcade games away from school?'. Response categories for each item included 1) none, 2) 1 hour, 3) 2 hours, 4) 3 hours, 5) 4 hours, 6) 5 hours, 7) 6 hours or more. Total sedentary behavior was defined as the sum of these 3 sedentary behaviors, which means there is a minimum of zero and a maximum of 18 hours. Other variables from SPAN were African-American (1 = yes, 0 = no), Hispanic (1 = yes, 0 = no), speaks Spanish at home (1 = yes, 0 = no), age, speaks language other than Spanish or English at home (1 = yes, 0 = no). The percentage disadvantaged at the school comes from the Texas Education Association (TEA) [33].

The crime variables came from two sources. First, we used the U.S. Department of Justice, Office of Justice Programs, Bureau of Justice Statistics data for the years 2000 through 2005 [34]. The community crime statistics were robberies, all violent crimes, murders, assaults, property crimes, rapes, burglaries, larcenies, motor vehicle thefts, and sexual offenders living in the police department where the school is located. We included the average crime rates per hundred population for the years 2000 to 2005 in each police department. Police departments correspond to cities over 100,000 in population. Note that our six-year average meant that recent crimes "count" the same as crimes five years ago. Parents in a normally safe community suffering a recent up tick in crime may not bring their children inside, especially if the offending person(s) have been apprehended. Additionally, if criminal activity in an area declines after a long period of high crime activity, parents would likely remain reluctant to allow their children to play outside in the near term. Thus, criminal activity from five to six years ago should also count because it will influence parent's current decisions about their children's activity. Second, we included the number of sexual offenders, as of September 2007, living in the zip code address of the school [35]. These data are listed by the Texas Department of Public Safety under the Texas Sex Offender Registration Program. The sexual offenders were per 100 population in the zip code of the school. We did not have the zip code of the student.

Overall, there were 160 schools with an average of 49.4 students per school. There were 111 unique zip codes for the schools. Schools were linked to 39 police districts reporting crime statistics.

All estimates and statistical tests were performed taking into account SPAN's sample design features. STATA (version 8.0, StataCorp LP, College Station, Texas) was used to analyze the data. Weighted means, population standard errors, and proportions for demographic characteristics were computed. Adjusted incremental relative risk (IRRs) for indoor sedentary behavior associated with crime levels and their corresponding 95% confidence intervals (95% CI) were calculated using sampling weighted Poisson regression models for each sedentary behavior measure (count of hours during the previous day). Adjustment variables were sex, African-American, age, percentage disadvantaged in school, Hispanic, Spanish spoken at home and language other than Spanish or English spoken at home. An a level of 0.05 was established a priori as the probability of incurring a type I error.

## Results

The mean age of 4^*th *^grade students was 9.7 and was similar for boys and girls (Table 1 [See additional file 1]). Fifty-one percent of the 4^*th *^graders were male, and 45 percent were Hispanic and approximately 25 percent spoke Spanish at home. Boys spent more hours in sedentary behavior per day than girls, spending 5.2 hours per day watching TV (0.206 standard error), playing video games, or using the computer compared to 3.6 hours per day (0.098 standard error) for girls. Boys watched slightly more TV than girls, but TV made up over half of the sedentary behavior for girls. Boys played video games almost three times more than girls, spending approximately two hours a day. Both boys and girls spent approximately an hour per day on the computer. Note that all sedentary behavior was outside of school.

Table 1 [See additional file 1] lists all of the per 100 population crime rates, averaged from 2000 to 2005 in the city of the school. Sexual offenders in the zip code of the school were included in per capita form.

Incremental relative risks for total daily hours of video game playing, adjusted by sex, African-American, Hispanic, speaks Spanish at home, age, speaks language other than Spanish or English at home and percentage disadvantaged at the school, were reported in Table 2 [See additional file 1]. For the total sample, sex offenders per capita was associated with increased video game playing (IRR 2.558, 95% CL 1.177, 5.560). For boys, sex offenders per capita was associated with increased video game playing (IRR 2.347, 95% CL 1.270, 4.337) as were burglaries per 100 population (IRR 1.246, 95% CL 1.054, 1.472). For girls, crime was not associated with increased video game playing. The magnitude of the association between the number of sexual offenders per capita and video game playing is quite large. When evaluated at the mean level of sex offenders per capita, a one percentage point increase in per capita sex offenders would be associated with an increase of approximately 1.2 hours of video game playing per day. For boys, a one percentage point increase in per capita sex offenders would be associated with an increase of approximately 1.7 hours of video game playing per day. The magnitude of the association between burglaries for boys were much lower. When evaluated at the mean level of burglaries per 100 population, a 10 percentage point increase would be associated with an increase of approximately 0.044 hours, or 2 minutes and 40 seconds, of video game playing per day.

Incremental relative risks for total daily hours of computer use, adjusted by sex, African-American, Hispanic, speaks Spanish at home, age, speaks language other than Spanish and English at home and percentage disadvantaged at the school, were reported in Table 2 [See additional file 1]. For the total sample and boys, none of the crime variables was associated with increased computer use. For girls, sex offenders per capita was protective against computer use (IRR 0.394, 95% CL 0.161, 0.964). When evaluated at the mean level of sex offenders per capita, a one percentage point increase in per capita sex offenders would be associated with a decrease of approximately 48 minutes of computer use by girls per day.

Incremental relative risks for total daily hours of TV viewing, adjusted by sex, African-American, His-panic, age, speaks language other than Spanish and English at home and percentage disadvantaged at the school, were reported in Table 2 [See additional file 1]. For girls, burglaries and hours of television watching were positively associated (IRR 1.186, 95% CL 1.038, 1.356) as were larcenies (IRR 1.051, 95% CL 1.001, 1.104). When evaluated at the mean level of burglaries per 100 population, a 10 percentage point increase would be associated with an increase of approximately 0.031 hours, or approximately 2 minutes of television viewing per day. When evaluated at the mean level of larcenies per 100 population, a 10 percentage point increase would be associated with an increase of approximately 0.032 hours, or approximately 54 seconds of television viewing per day.

## Discussion

Our results showed that 4^*th *^grade boys played more hours of video games in communities with increased levels of larceny and burglary crimes as well as for increased per capita sexual offenders; 4^*th *^grade boys played more hours of video games for increased levels of per capita sexual offenders; 4^*th *^grade girls were more likely to watch television in areas with more burglaries and larcenies. Computer use was negatively associated with per capita sexual offenders among girls. However, it was not significant for boys, even though boys used the computer more hours per day than girls. Community crime rates were not associated with total indoor sedentary behavior.

Our results revealed that community crime rates affected whether girls and boys engaged in indoor sedentary behavior, although the types of activities differed by gender. We had expected the crime rates to affect indoor sedentary behavior for boys more than girls because the latter have traditionally played, and continue to play, indoors more than boys [36]. So while it is not surprising to see girls using leisure technologies, the effect of crime in neighborhoods is more surprising due to the smaller time spent playing outdoors before the development of leisure technologies. It is likely that time spent with leisure technologies is being substituted for the small outdoor play time alloted to girls relative to boys, especially in high-crime neighborhoods.

Sexual offenders per capita were protective against computer use for 4^*th *^grade girls. Parents in these communities may be reluctant to allow their girls to be potentially exposed to online sexual advances.

Sexual crimes, particularly those against children, are highly emotional, which may partly explain the greater influence on indoor sedentary behavior in our sample. However, there is another potential explanation. Perceptions of crime may not coincide with actual crime rates in an area [37-39]. By Texas law, jurisdictions can promote the presence of sexual offenders through mailings, advertisements, and the inter-net [40]. Therefore, while the public may overstate the danger posed by sexual offenders, their knowledge of the presence of offenders may be more accurate than their knowledge of the prevalence of other crime measures.

New technologies which complement sedentary behavior may be having subtle effects on outdoor play, even for children without access to technologies promoting sedentary behavior. For instance, there may be "threshold effects" associated with children's outdoor play which have been negatively affected by the availability of new technologies. If new video game technologies have drawn children indoors, especially in neighborhoods with high crime rates, thresholds may not be met. Then, even children without video games and cable TV have less opportunity to play outdoors because many of their peers are indoors. Further, with fewer children playing outside, perhaps parents will not allow their children to play freely outside-parents may believe there is safety in numbers in regards to criminal activity. For all of these reasons, today's children may face decreased numbers of playmates available outdoors.

If parental fear of crime in their communities, in conjunction with the development of inexpensive leisure activity technologies for children, is tipping the balance towards indoor sedentary play, then there are important policy implications. Crime will never be completely eliminated; the development of video games, computers, and ever more television programming will continue apace. However, children still likely prefer to socialize, play team sports and to roam free outdoors. Therefore, after school programs promoting physical activity which feature adult supervision should be expanded because they will ease parental fears about crime. Team sports, which in years past would form spontaneously, must be organized by parents. Indeed, the secular trends are towards more time spent in supervised sports and activities among children, perhaps because of safety concerns due to crime [41]. As noted earlier, access to safe play environments has been shown to be associated with increased physical activity [27]

Because the SPAN survey takes place during the school year, our results likely underestimate the true association between crime and indoor sedentary activity because more time is spent indoors in the summer. In Texas, where this study takes place, the percentage of houses with air conditioning has reached 90 percent [22]. Heat-related mortality declined significantly from the 1960s to the 1990s due to air conditioning, especially in the Southeast and Southwest [22]. Therefore, indoor play has become preferable relative to outdoor play due to temperatures in the summer.

Our results have limitations. Of course, a large cohort study would have been preferred to compare changes in crime and their effects on physical activity. Another problem is that computer use for homework cannot be excluded from our total sedentary and our total hours of computer use measure. It is possible that computers are used for homework in low crime areas. Further, the number of sexual offenders per capita is in the zip code area of the school, but it may be different in the residential area where the children live. Our crime statistics are likely imperfect estimates of neighborhood crime. Our crime rates for robberies, all violent crimes, murders, assaults, property crimes, rapes, burglaries, larcenies and motor vehicle thefts, are estimated by city police department jurisdiction. In several instances, two or more schools are located within the same police department jurisdiction. However in almost all cases, schools had unique per capita sexual offender estimates.

Our controls for socioeconomic status are imperfect because of the diffculty in asking children questions related to income. Further, income itself has conflicting effects on indoor sedentary behavior. Although the technologies are more affordable, it is possible that many wealthy suburban communities have gated residential lots which would lead children to substitute outdoor play for indoor sedentary activity. It has been long noted in the area of urban economics that wealthier families live in the suburban areas with large lots [42,43]. However, we do not have information on residential lot size nor whether the child's yard is gated, both of which likely are conducive to outdoor play.

Children most likely enjoy outdoor activities. Our results show that parental fears of crime may be as important as child preferences for sedentary leisure. Therefore, policies which alleviate parental fears may increase childhood physical activity.

## Competing interests

The authors declare that they have no competing interests.

## Supplementary Material

Additional file 1 Tables 1 and 2. Descriptive statistics and regression results. Click here for file
