# Supplementary material for: Crime rates and sedentary behavior among 4th grade Texas school children
Source: Int J Behav Nutr Phys Act. 2008 May 14;5:28. doi: 10.1186/1479-5868-5-28 (PMC2412913; doi:10.1186/1479-5868-5-28)
Supplement: Additional file 1 — Tables 1 and 2. Descriptive statistics and regression results. [file 1479-5868-5-28-S1.doc]

| Table 1: Sample Characteristics/Descriptive Statistics | | | | | | |
| --- | --- | --- | --- | --- | --- | --- |
|  | Total Sample | | Boys | | Girls | |
|  | n=7,907 | | n=3,956 | | n=3,951 | |
|  | N=248,838 | | N=127,296 | | N=121,542 | |
| *Student Demographics* | Proportion | Standard Err | Proportion | Standard Err | Proportion | Standard Err |
| Boy (1=yes, 0=no) | 0.512 | 0.019 |  |  |  |  |
| African-American (1=yes, 0=no) | 0.134 | 0.019 | 0.135 | 0.023 | 0.134 | 0.017 |
| Hispanic (1=yes, 0=no) | 0.446 | 0.035 | 0.445 | 0.035 | 0.447 | 0.038 |
| Age (mean) | 9.742 | 0.039 | 9.772 | 0.045 | 9.711 | 0.037 |
| Spanish Language at Home (1=yes, 0=no) | 0.252 | 0.049 | 0.252 | 0.051 | 0.252 | 0.049 |
| Other language at Home (1=yes, 0=no) | 0.016 | 0.007 | 0.017 | 0.014 | 0.014 | 0.009 |
| Percentage Disadvantaged at School | 0.632 | 0.035 | 0.618 | 0.032 | 0.647 | 0.039 |
| *Student Sedentary Behavior* | Mean | Standard Err | Mean | Standard Err | Mean | Standard Err |
| TV (Hours Yesterday) | 2.065 | 0.059 | 2.243 | 0.089 | 1.879 | 0.060 |
| Computer (Daily Average # of Hours) | 0.933 | 0.051 | 0.948 | 0.075 | 0.917 | 0.039 |
| Video Games (Daily Average # of Hours) | 1.443 | 0.084 | 2.057 | 0.206 | 0.799 | 0.062 |
| Sedentary Behavior (Hours, Daily Average) | 4.444 | 0.138 | 5.225 | 0.206 | 3.611 | 0.098 |
| *Community Crime* | | | | | | |
| Robberies per 100 population* | 0.206 | 0.026 |  |  |  |  |
| Violent Crimes per 100 population* | 0.679 | 0.056 |  |  |  |  |
| Murders per 100 population* | 0.007 | 0.038 |  |  |  |  |
| Assaults per 100 population* | 0.419 | 0.031 |  |  |  |  |
| Property Crimes per 100 population* | 5.917 | 0.255 |  |  |  |  |
| Rapes per 100 population* | 0.047 | 0.313 |  |  |  |  |
| Burglaries per 100 population* | 1.124 | 0.044 |  |  |  |  |
| Larcenies per 100 population* | 4.211 | 0.159 |  |  |  |  |
| Motor Vehicle Thefts per 100 population* | 0.582 | 0.063 |  |  |  |  |
| Sex offenders per 100 population** | 0.0014 | 0.0001 |  |  |  |  |
| *Crime rates in the city where the school is located. Source: Bureau of Justice Statistics. | | | | |  |  |
| **Per capita sex offenders in the zip code where the school is located. Source: Texas Department of Public Safety, Sex | | | | | | |
| Offender Registration Program |  |  |  |  |  |  |

Table 2 Continued

*Adjusted by sex, African-American, Hispanic, speaks Spanish at home, age, speaks language other than Spanish and English at home and percentage disadvantaged at the school

**Crime rates in the city where the school is located. Source: Bureau of Justice Statistics.

***Per capita sex offenders in the zip code where the school is located. Source: Texas Department of Public Safety, Sex Offender Registration Program
